# Unnecessary reliance on multilevel modelling to analyse nested data in neuroscience: When a traditional summary-statistics approach suffices

**DOI:** 10.1016/j.crneur.2021.100024

**Published:** 2021-11-17

**Authors:** Carolyn Beth McNabb, Kou Murayama

**Affiliations:** a School of Psychology and Clinical Language Sciences, University of Reading, Early Gate, Reading, RG6 7BE, United Kingdom; b Hector Research Institute of Education Sciences and Psychology, University of Tübingen, Europastraße 6, 72072 Tübingen, Germany; c Research Institute, Kochi University of Technology, Tosayamada, Kami City, 782-8502, Kochi, Japan

**Keywords:** Hierarchical linear model, Mixed model, t-test, Clustering

## Abstract

Nested data structures create statistical dependence that influences the effective sample size and statistical power of a study. Several methods are available for dealing with nested data, including the summary-statistics approach and multilevel modelling (MLM). Recent publications have heralded MLM as the best method for analysing nested data, claiming benefits in power over summary-statistics approaches (e.g., the *t*-test). However, when cluster size is equal, these approaches are mathematically equivalent. We conducted statistical simulations demonstrating equivalence of MLM and summary-statistics approaches for analysing nested data and provide supportive cases for the utility of the conventional summary-statistics approach in nested experiments. Using statistical simulations, we demonstrate that losses in power in the summary-statistics approach discussed in the previous literature are unsubstantiated. We also show that MLM sometimes suffers from frequent singular fit errors, especially when intraclass correlation is low. There are indeed many situations in which MLM is more appropriate and desirable, but researchers should be aware of the possibility that simpler analysis (i.e., summary-statistics approach) does an equally good or even better job in some situations.

## Highlights

• Multilevel modelling is not superior to summary statistics when clusters are balanced.
• Summary statistics provides benefits when singular fit errors occur.
• Previous reports of superior performance with multilevel modelling are not supported.

## Graphical abstract

**Figure:**
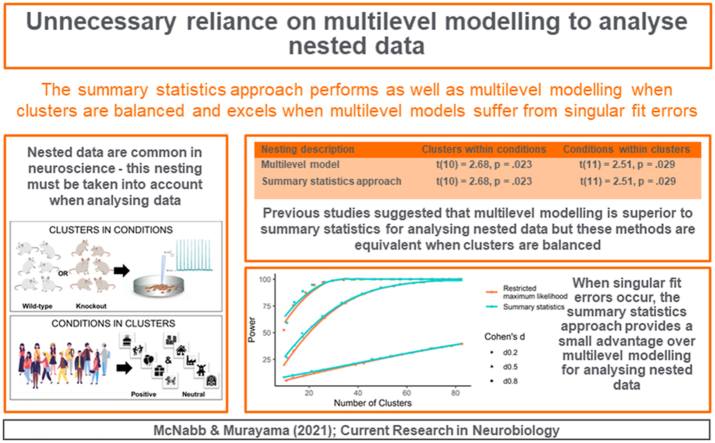

Nested (hierarchical) data structures are common in neuroscience and create dependence in the data that influences the effective sample size and statistical power of a study (Moen et al., 2016; Zyzanski et al., 2004). Two main types of nesting occur commonly in this field: 1) nesting of clusters within conditions (Fig. 1a and c; e.g., an experimenter compares cells from 6 wild-type and 6 knockout mice, taking 10 electrophysiology recordings from each mouse) and 2) nesting of conditions within clusters (Fig. 1b and d; e.g., 12 participants in a psychology experiment respond to 5 stimuli from a neutral condition and 5 stimuli from a positive condition). In their simplest forms, these methods may be thought of as “between-subjects” and “within-subjects” designs, respectively (provided each subject provides multiple data points) - though these representations may not hold up in more complicated study designs.

**Fig. 1:**
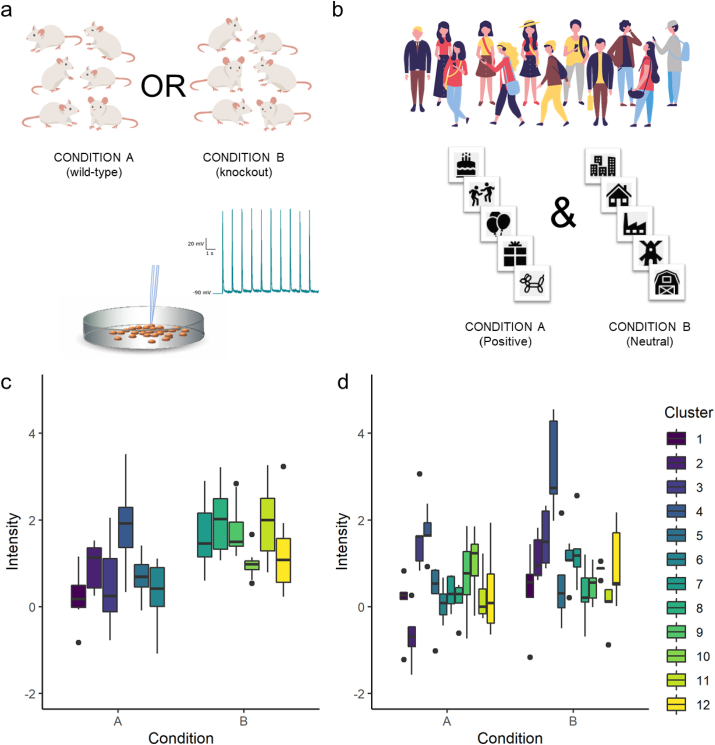
Examples of nested data where clusters are nested within conditions (a and c) and conditions are nested within clusters (b and d). Images adapted from freepik.com and vecteezy.com.

Ignoring the nested structure of hierarchical data (i.e., analysing data points as if they were independent from one another - known as pooling) can result in inflated Type I errors. This is because nested data possess some level of redundancy. In scenario 1) above, for instance, there are 120 data points from 12 subjects, but because data points *within* a mouse are more similar than data points *between* mice, the effective information contained in the data fall short of the information that would have been obtained from 120 independent data points. As a result, if we analysed the data using an independent samples *t*-test with *N* = 120, we would arbitrarily have over-estimated *N*. This subsequently under-estimates standard errors and inflates Type I error rates. Fortunately, there are several methods available for taking this nested structure into account.

Among them, multilevel modelling (MLM) has been advocated as the best way to analyse nested data in neuroscience (Galbraith et al., 2010), as it controls for Type I errors while ensuring high statistical power. These features have made MLM increasingly popular with neuroscientists analysing this type of data. However, the challenges associated with MLM, including model convergence and singular fit issues, can limit its applicability. Furthermore, and importantly, the conditions under which multilevel models have outperformed simple statistical methods (such as the *t*-test) and the associated gains in power reported in previous influential literature (Aarts et al., 2014, 2015) are based on statistical simulations that use a method of standard error estimation that is inappropriate (discussed in detail in section 2).

In fact, as we will establish, conventional analysis using the summary-statistics approach is mathematically equivalent to MLM, provided that the data are not cross-nested and that cluster size is equal (i.e., there is no gain in power with MLM in such cases). Furthermore, the sufficient summary-statistics approach, which accounts for variance within clusters, is almost equivalent to MLM under conditions where cluster size does vary. This equivalence has been established in statistical literature (Beckmann et al., 2003; Dowding and Haufe, 2018; Murayama et al., *in press*) and made use of in popular neuroimaging software such as FMRIB Software Library – FSL (Beckmann et al., 2003) and Statistical Parametric Mapping – SPM (Mumford and Nichols, 2009). However, the merit of the summary-statistics approach has not been well recognized in neuroscience beyond that particular context. Here we provide support for the utility of the conventional summary-statistics approach in nested experiments and show that losses in power attributed to the use of summary-statistics in previous publications (Aarts et al., 2014, 2015) are unsubstantiated. This manuscript will primarily address data where the predictor variable is binary and sample size is balanced between groups (i.e., those scenarios introduced in Fig. 1) for ease of understanding and given the popularity of this design in the field. However, some experiments may require more complicated study designs, for which the two methods (summary-statistics approach and MLM) will not always be equivalent. These scenarios are addressed in section 5.

## 1 Equivalence of multilevel modelling and summary-statistics approach for nested data with equal cluster size

The summary-statistics approach comprises two steps: 1) compute the mean (i.e., summary-statistics) for each cluster from the nested data; 2) apply a statistical test (e.g., *t*-test) to the cluster means (i.e., the mean of all observations in each cluster) using clusters as the unit of analysis. Because cluster means are independent from each other, the second step does not suffer from the data dependency issue. If clusters are nested within conditions (Fig. 1a), cluster means in condition A can be compared with the cluster means in condition B using an independent two-sample *t*-test (with *N* = 6 for each condition in the example). For data where conditions are nested within clusters (Fig. 1b), the cluster means in condition A and cluster means in condition B can be compared using a paired *t*-test (with *N* = 12 in the example). Aside from the pooling approach described earlier, which is statistically inappropriate, this is the most conventional method to analyse nested data.

On the other hand, MLM analyses all the data at once. In the MLM approach, the main effect of condition is considered the *fixed effect*, while the nested nature of the data is represented by the *random effects*. Random effects quantify the degree of between-cluster variation. In the context of the current examples, there are two components: *Random intercepts*, which reflect by-cluster variation of the intercepts and *random slopes*, which reflect by-cluster variation of the effects of the condition. While we need to consider both random intercepts and slopes if conditions are nested within clusters (scenario 2), we only need to consider random intercepts if clusters are nested within conditions (scenario 1), because it is impossible to estimate by-cluster variation of the condition effects (i.e., each cluster is assigned to only one of the conditions).

If we were to write these MLM equations for the *lmer* package in *R*, they would look like this:

(1) $$\text{Scenario }1\text{: }lmer\left(Intensity\;\sim \;Condition\;+\;(1\;|\;Cluster),\;data\right)$$

(2) $$\text{Scenario }2\text{: }lmer\left(Intensity\;\sim \;Condition\;+\;\left(1\;+\;Condition\;|\;Cluster\right),\;data\right)$$

Critically, for both designs, the summary-statistics approach and MLM produce identical results when cluster sizes are equal (for mathematical proof, see Murayama et al. (*in press*)). To demonstrate the statistical equivalence of these two approaches, we randomly simulated two datasets in which clusters were nested within conditions (Dataset A, see Fig. 1a for visual example of data) or conditions were nested within clusters (Dataset B, and see Fig. 1b for visual example). Data were simulated using R version 4.0.1 (2020-06-06). For the current example, cluster size was set to 10 and number of clusters was set to 12. We then compared conditions using MLM and summary-statistics analysis (i.e., *t*-test for cluster means). Results are presented in Table 1. For both types of hierarchical data, *t* values were equal for multilevel and summary-statistics approaches, indicating that summary-statistics perform just as well as MLM for these data. It is also worth noting that the equivalence generally holds however many predictors researchers included in the model. For more detailed discussion on the conditions for the equivalence, please see Murayama et al. (*in press*).

**Table 1:** Equivalence of multilevel model and summary-statistics approaches for analysing data with equal cluster sizes.

	Dataset A	Dataset B
Nesting description	Clusters within conditions	Conditions within clusters

**Multilevel model**	Intensity ∼ Condition + (1 | Cluster)	Intensity ∼ Condition + (1 + Condition | Cluster)
*Fixed effects (SE)*		
Intercept	.77 (.22) *t*(10) = 3.54, *p* = .005	.49 (.20) *t*(11) = 2.52, *p* = .028
Slope	.83 (.31) ***t*(10) = 2.68, *p* = .023**	.49 (.20) ***t*(11) = 2.51, *p* = .029**
*Random effects*		
Intercept variance	.23	.35
Slope variance		.25
Correlation		-.01
Within cluster (residual) variance	.51	.53
**Summary-statistics approach**		
Two sample *t*-test	***t*(10) = 2.68, *p* = .023**	
Paired samples *t*-test		***t*(11) = 2.51, *p* = .029**

## 2 Debunking the myth of power loss with the summary-statistics approach

Aarts and colleagues suggested that, for hierarchical data where clusters are nested within conditions (such as Dataset A/Scenario 1), MLM affords increased statistical power compared to the summary-statistics approach when the number of clusters is few (see Fig. S1 for replication of Aarts et al. power loss), while keeping Type I error rate to the nominal level (Aarts et al., 2014). In their paper, Aarts et al. simulated nested datasets with (equal) cluster size = 5 and number of clusters (sample size) ranging between 10 and 82 (10,000 simulations at each sample size). Comparing *t* tests conducted on cluster-based means with MLM, they claimed a 40% loss of statistical power with the summary-statistics approach, depending on cluster size and intra-class correlation (Aarts et al., 2014). This “power loss” has since been promoted and reiterated in the literature (Saravanan et al., 2020), contributing to the increased popularity of MLM and other advanced statistical techniques in neuroscience. However, as we discussed above, when cluster sizes are equal, these two approaches are actually mathematically equivalent – there is in fact no gain in statistical power with MLM when data are balanced.

**Fig. 2:**
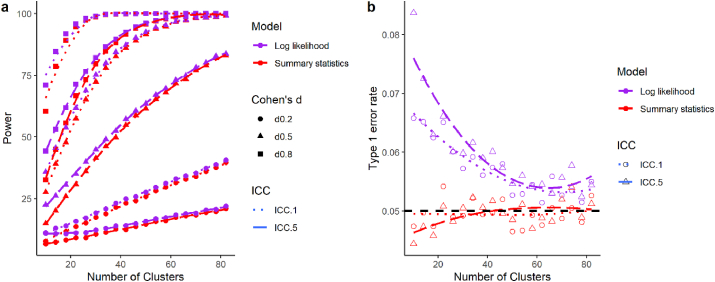
Increased power and associated increases in Type I error rate with use of multilevel modelling (MLM) with log-likelihood test (purple) compared with summary-statistics approach (red) when number of clusters is few. Power (a) and Type I error rate (b) are shown for all 10,000 simulations, including data that resulted in singular fit or convergence warnings with MLM. Data are shown for small (solid circle), medium (solid triangle) and large (solid square) effect sizes (Cohen’s d) and intra-class correlation (ICC) corresponding to the majority of total variance being due to within-group variance (ICC = .1, dotted line) and more total variance being due to between-group variance (ICC = .5, dashed line). (For interpretation of the references to colour in this figure legend, the reader is referred to the Web version of this article.)

Why then, did Aarts and colleagues’ simulation show increased statistical power with MLM compared to the summary-statistics approach? This is due to the method of statistical test they used for the MLM. Data in Aarts et al.’s simulations were analysed using the maximum likelihood (ML) estimation and log-likelihood ratio test using z statistics (see Fig. 2). The log-likelihood test is asymptotically correct, meaning that this is an appropriate statistical test when cluster size tends towards infinity, provided all the assumptions are met and model is correctly specified. However, this method is known to be anticonservative when sample size is small (Manor and Zucker, 2004). Consequently, as the power benefit of MLM increases with fewer clusters, so too does the Type I error rate. Although Aarts et al. (2015) did not report the Type I error rate in their paper (Type I error rate was reported in a separate simulation that focused only on cases in which number of clusters were reasonably large), this was exactly what happened - increased power with MLM simply reflected increased Type I error rate. Conversely, the more common testing method for fixed-effects is to use a *t*-test statistic based on restricted maximum likelihood estimation (REML; the default option for most statistical packages such as lme4 in R, SAS, SPSS, and HLM). As *t*-test statistics take into account sample size by adjusting the distribution according to degrees of freedom^1^ (df), the *t*-test can better control for Type I error rate. Although REML estimates of standard errors also have some underestimation bias with small samples, this is less of an issue, especially when data are balanced (see our simulation results below).

It is also worth noting that Aarts et al. (2015) defined loss in power as [(Power_MLM_-Power_Summary-statistics approach_)/Power_MLM_]*100. In other words, “power loss” is the proportion of power reduced, not the actual difference in power estimates. Consequently, the loss of power may appear big when overall power is low (e.g., if the power from MLM is 8% and that from summary-statistics approach is 7%, the actual difference is only 1%, but this is described as “12.5% loss in power”). To provide a more accurate impression of power difference, the current manuscript shows power for each approach (MLM and the summary-statistics approach) separately.

To illustrate our point, we conducted a simulation to estimate statistical power and Type I error rate using the log-likelihood ratio test, REML (*t*-test) and summary-statistics approach (simulations conducted in R version 4.0.2 (2020-06-22; R scripts and simulated data are available on our OSF page - https://osf.io/w6unc/). When cluster size is small, consistent with the simulation results by Aarts et al. (2014), the log-likelihood ratio test shows advantages in statistical power (Fig. 2a). Note, though, that the actual difference in power is very small. However, this test suffers from inflated Type I error rate above the nominal alpha (5%) level (Fig. 2b). Conversely, REML (*t*-test; Fig. S2) and the summary-statistics approach (Fig. 2), which showed identical results in the absence of singular fit or convergence errors, appropriately control for Type I error rate across all simulation conditions, even when sample size is small.

## 3 Cost of multilevel modelling

**Fig. 3:**
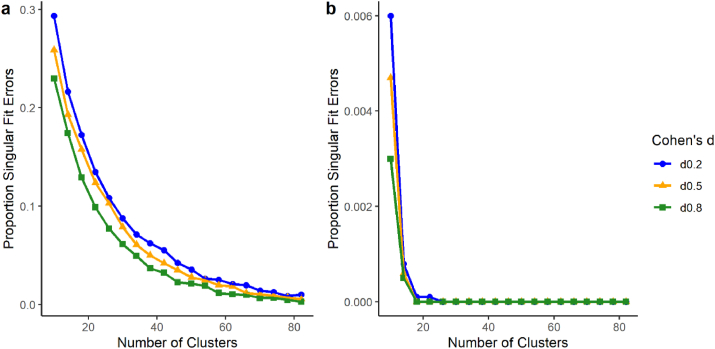
Proportion of total simulations resulting in singular fit errors when using restricted maximum likelihood estimation (REML) under conditions of varying effect size (Cohen’s *d*) and intra-class correlation (ICC). Plots show the proportion of simulations resulting in singular fits when a) ICC = .1 and b) ICC = .5.

When data are balanced, the summary-statistics approach and MLM with REML provide identical results, but the MLM remains susceptible to other issues, such as singular fits (when the estimated variance-covariance matrix is rank-deficient) and convergence warnings (when the model never settles on a global - or even local - optimum). Researchers frequently come across such error messages in MLM (Brauer and Curtin, 2018). Within our simulated datasets, singular fit errors occurred at a much higher frequency than convergence warnings.^2^ Singular fit errors were more frequent when intra-class correlation (ICC) was low (i.e., the majority of total variance is due to within-group variance) than when ICC was higher (i.e., more total variance is attributed to between-group variance). This was especially evident when sample size was small - singular fits occurred in over 20% of models when the ICC was 0.1 (Fig. 3a) but in less than 0.7% of models when ICC was 0.5 (Fig. 3b). Furthermore, lower ICC was associated with singular fits even when sample size was very large (82 clusters of 5 observations each), whereas singular fits did not occur at larger sample sizes when ICC was higher. McNeish and Bauer (2020) also showed that singular fit errors are more frequent when cluster size is small, and this issue has been echoed throughout the literature (Lorenzo-Seva and Ferrando, 2020; van de Schoot and Miocević, 2020). It is worth noting that these errors occurred even under the ideal situation afforded by the statistical simulation - when models were correctly specified (using REML) and all assumptions of MLM were met.

Because singular fit errors can occur due to a near-zero amount of variance explained by a random effect, some researchers recommend removing random effects with near-zero variance from the model prior to testing fixed effects (e.g., using information-theoretic criteria, such as Akaike Information Criteria or Bayesian Information Criteria (Zuur et al., 2009) or principle components analysis (Bates et al., 2018)). On the one hand, removing random effects make the model more parsimonious and powerful. At the same time, however, if the random effect actually exists (which we cannot definitely know from the data, even based on model comparison and selection), removing it could underestimate standard errors, inflating Type I error rate.^3^ Barr et al. (2013) are particularly explicit about this risk.

Importantly – researchers are warned against removing random slopes from the model when conditions are nested within clusters (scenario 2). Barr et al. (2013) note that “[f]or designs including within-subjects (or within-items) manipulations, random-intercepts-only [linear mixed effects models (MLM)] can have catastrophically high Type I error rates, regardless of how p-values are computed from them”. Although this is still a matter of debate (Barr et al., 2013; Bates et al., 2018) we would caution readers to be aware of (and address) these risks if employing such methods.

Importantly, singular fit and convergence errors do not happen at random. Consequently, the fact that these errors did occur implies that there is a certain systematic property in the observed data, making it impossible to control for Type I and Type II error rates in an appropriate manner. More specifically, when we exclude the cases of singular fit and convergence errors in the simulation with REML (the majority of errors were singular fit errors as noted above), Type I error rate was actually lower than 5% (Fig. S2b). This slight reduction in Type I error rate was associated with a small **loss** in power of up to 6% (REML-summary statistics approach) compared with the summary-statistics approach (Fig. S2a).

These results highlight the limitations of multilevel models for analysing nested data, especially when sample size (number of clusters) is small. Moreover, the frequency of encountering an estimation issue in real datasets may be even higher than what we observe in our simulations due to the noisy nature of real data. Conversely, a summary-statistics approach does not suffer from such estimation issues, as it does not use an iteration procedure; and it will always provide a reliable test statistic.

The summary-statistics approach is also flexible to the choice of summary statistic. In some settings (e.g., when measuring reaction or latency times in behavioural data, where data typically exhibit a skewed distribution), the mean response may not be the most appropriate univariate statistic to describe the data. In such instances, researchers may wish to use the bias-corrected^4^ median response time as it may be a more robust representation of a participant’s typical response compared with the mean. Using the summary-statistics approach, researchers have the flexibility to choose the most representative summary statistic for the type of data being analysed - though such a plan should be made in advance (e.g., preregistering analysis methods).

## 4 Some complicated cases: Nested data with variable cluster size or continuous predictors

We have demonstrated that the summary-statistics approach is equivalent to MLM when cluster size is equal but actually these approaches produce very similar results even when cluster size varies moderately across clusters (see Table S1, but also Murayama et al. (*in press*)), meaning that even when there are missing data for certain clusters or conditions, this may not impact the outcomes of the summary-statistics approach to a great degree. When cluster size varies substantially, however, researchers can employ the *sufficient* summary-statistics approach. This is especially useful if issues with MLM arise due to singular fits or convergence errors.

The sufficient summary-statistics approach (Dowding and Haufe, 2018) takes into account both the cluster mean and the intra-cluster variance. When different conditions exist within clusters (e.g., Fig. 1b/Scenario 2), researchers can compute the effect size (including raw mean differences) and its standard error between the conditions, and integrate the effect size across the clusters by weighting a cluster’s mean according to the inverse of that cluster’s variance. This adjustment effectively ‘downplays’ the contribution of outlier clusters, which could otherwise significantly affect the estimation of the inter-cluster variance. This is effectively how a meta-analysis works - taking the effect sizes and their variances across multiple studies to derive the *integrated* mean effect size. When conditions are allocated between clusters (e.g., Fig. 1a/Scenario 1), researchers can compute the mean and standard errors for each cluster, and then conduct a so-called moderator analysis or meta-regression analysis (Borenstein et al., 2011) to examine the effect of condition (binary coded). This method takes into account the sampling variability of the cluster mean using a weighting procedure. The sufficient summary-statistics approach is often called the two-step approach or the variance-known model in the context of MLM (Leoni, 2009; Raudenbush and Bryk, 2002). There are different methods for weighting, but generally speaking, both MLM and the sufficient summary-statistics approach would produce asymptotically equivalent estimates (Achen, 2005). The usefulness of the sufficient summary-statistics approach has sporadically but long been discussed in other fields, e.g., econometrics (Saxonhouse, 1976), and the principle has already been adopted for statistical parametric mapping in neuroimaging analysis (Beckmann et al., 2003). But the neuroscience community is generally unaware of the utility of this approach - for an exception, see Dowding and Haufe (2018). This type of analysis can be easily conducted by using a meta-analysis package (e.g., metafor package in R (Viechtbauer, 2010)).

The current paper has focused on a case in which predictors are binary (i.e., comparison of conditions). But consider a different case: what about a model having continuous predictors? MLM can effectively deal with this situation, but we can also use the summary-statistics approach. Specifically, we can conduct a regression analysis for each level 2 unit (e.g., computing a regression coefficient for each participant in an experiment with multiple trials) and then conduct a one sample *t*-test on the regression (i.e., beta) coefficients from all level 2 units, in order to examine whether the regression coefficient is significantly different from zero on average. This analysis provides similar results to MLM (Lorch and Myers, 1990; Murayama et al., *in press*) and is the approach commonly taken by neuroimaging analysis software (e.g., SPM (Mumford and Nichols, 2009)). Furthermore, by using the sufficient summary-statistics approach with standard errors of regression coefficients as the source of weights, we can obtain essentially the same results as with MLM (Beckmann et al., 2003). This approach is also employed by neuroimaging analysis software (e.g., FSL (Beckmann et al., 2003)). Having continuous predictors does not stop researchers from having the options of these two approaches.

As indicated here, for certain instances, the multilevel model and summary-statistics approaches are equally appropriate tools. A big advantage of the sufficient summary-statistics approach is that it is computationally economical and does not suffer from singularity or convergence errors. Therefore, it may sometimes be deemed a more practically favourable alternative when analysing nested data (Dowding and Haufe, 2018).

## 5 Complex study designs for which multilevel models are optimal

There are of course many instances where the summary-statistics approach and sufficient summary-statistics approach are less well equipped to analyse data. For example, summary-statistics approaches cannot be used to directly estimate variance components. The summary-statistics approach also cannot deal with data that have crossed random effects (Baayen et al., 2008) (when the random factors themselves are crossed, not nested) and cannot be applied to a model when there are more than two levels (e.g., if Scenario 1, above, included multiple litters of wildtype and knockout mice, for which we might expect there to be different effects depending on litter, and as before, multiple cells from each mouse were analysed in the experiment). In those situations, MLM is more suitable.

The summary-statistics approach and sufficient summary-statistics approach also cannot address cases in which researchers are interested in non-normal data --- e.g., when dealing with count or binary dependent variables. Note that when researchers are interested in comparing “proportions” between conditions (e.g., if for Scenario 1, we were interested in the proportion of cells able to generate an action potential for each condition – wildtype or knockout), this is essentially binary data. In such cases, an extension of multilevel modelling --- multilevel generalized linear modelling (also called generalized mixed-effects modelling) can be used. In essence, the summary-statistics approach is a good alternative to MLM but it has certain limitations.

## 6 Conclusion

Clustered data occur ubiquitously in neuroscience. While recent articles (Moen et al., 2016; Paternoster et al., 2018) have raised awareness of their existence and rightly herald the importance of taking hierarchical structure into account, there has become an over-reliance on MLM for analysing these types of data (McNeish et al., 2017). Here, we have demonstrated that the advantage of MLM for dealing with nested data is somewhat overstated in the literature. MLM does not offer benefits in power compared to the summary-statistics approach when cluster sizes are equal, as previously suggested (Aarts et al., 2014, 2015). In fact, when excluding singular fit errors, MLM with REML actually suffers from a slight loss in power compared with the more conventional summary-statistics approach. We have also discussed the robustness of the summary-statistics approach when cluster sizes vary moderately and discussed the utility of the sufficient summary-statistics approach when there are substantial variations in cluster size.

We are not suggesting that MLM is an inferior method by any means. In fact, MLM offers unique advantages to conventional methods, such as its capability to deal with complicated data and design as discussed above. Rather, we are suggesting that a conventional summary-statistics or sufficient summary-statistics approach can offer feasible alternative methods for dealing with nested data, and that these methods are robust to the limitations of MLM (i.e., rank-deficiency of the variance-covariance matrix or failure to find the global or local optimum). The summary-statistics approach is also incredibly useful for communicating and visualising results, even if the underlying analysis is MLM. Furthermore, using both summary-statistics and MLM approaches to analyse the same dataset can teach students about the underlying mathematics of these methods. It is not uncommon for MLM to be compared with the summary-statistics approach when MLM is taught to students or explained in the literature, but often, the summary-statistics approach is downplayed or deemed as inappropriate (Aarts et al., 2014, 2015). Researchers should be aware that, for some circumstances, the summary-statistics approach can actually do an equally good or even better job than MLM.

Exclusive reliance on MLM has been discouraged by others also. For example, McNeish et al. (2017) suggested cluster robust standard errors and generalized estimating equations as possible alternatives for analysing nested data. Likewise Datta and Satten (2005) developed a non-parametric approach using Rank-sum tests for when clustered data do not meet assumptions for parametric testing. Bootstrapping has been offered as another alternative approach (see preprint by Saravanan et al. (2020)). With many options available for analysing nested data, it is necessary for researchers to understand the risks and benefits of each method, rather than applying a blanket solution to all cases. However, as we have discussed, simple methods like the summary-statistics approach are often just as effective as more complex approaches, and do not suffer from the supposed losses in power that were previously shown.

## CRediT authorship contribution statement

**Carolyn Beth McNabb:** Conceptualization, Methodology, Formal analysis, Writing – original draft, revisions, Visualization. **Kou Murayama:** Conceptualization, Methodology, Writing – review & editing, Funding acquisition.

## Declaration of competing interest

The authors declare that they have no known competing financial interests or personal relationships that could have appeared to influence the work reported in this paper.

## Footnotes

1 The computation of degrees of freedom is still a matter of debate in mixed-effects modelling (for simulation studies, see Luke (2017) and Li and Redden (2015)). Several methods have been proposed for estimating effective degrees of freedom, including Satterthwaite’s method and Kenward-Roger’s method, which adjusts degrees of freedom/standard errors to account for small sample size (Kenward and Roger, 1997). Using the lmerTest package in R, researchers can choose between these methods (Kuznetsova et al., 2017). However, when data are balanced, these methods basically give the same degrees of freedom (Gelman, 2005). Therefore, researchers can simply use df = Nclusters – 2 (in cases where clusters are nested in conditions, e.g., scenario 1 in Fig. 1a) and df = Nclusters – 1 (in cases where conditions are nested in clusters, e.g., scenario 2 in Fig. 1b). *t*-test statistics for our simulations were calculated in lmerTest using the Satterthwaite approximation option (which returns the degrees of freedom equivalent to the equation above), but it actually does not matter which method we chose to use.

2 In our simulations, convergence warnings occurred at a relatively low rate and are not discussed further. However, researchers coming across these issues may wish to try different optimisers within the MLM, e.g., control = lmerControl(optimizer = "Nelder_Mead"), or via the optimx package with control = lmerControl(optimizer = 'optimx', optCtrl = list(method = 'L-BFGS-B')) (Nash and Varadhan, 2011). More recommendations for dealing with convergence issues can be found in (Brauer and Curtin, 2018).

3 Note that, although not perfect, there are also some potential solutions to the problem. For example, Brauer & Curtin provide a nice summary of potential remedies for convergence and singular fit errors in MLM (Brauer and Curtin, 2018). Among these, they indicate that, where there are singular fit errors, researchers can opt to retain only the random effect for the parameter for which they have a hypothesis (because this random effect is likely to be the source of the inflated Type I error rates one would obtain for the statistical test of that parameter). Another solution to the singular fit issue would be to use a factor analytic covariance structure (McNeish and Bauer, 2020). One could also use a Bayesian estimation method (using the R package brms, Bürkner (2017) for example) to avoid singular fit issues.

4 Miller (1991) warns that when reaction times (latencies in this case) are skewed, the median reaction time is biased and this bias is larger when the number of trials per condition is small. Based on this observation, Miller recommended against using median reaction time when comparing conditions with different trial numbers because the larger bias would cause conditions with fewer trials to appear slower (even if reaction time distribution is identical between conditions), thereby increasing Type I error rates. While Rousselet and Wilcox (2020) suggested that a bias-corrected median obtained using a bootstrap procedure could eliminate this bias problem, Miller (2020) – though in agreement that means and bias-corrected medians are both free of this bias and thus have acceptable Type I error rates - demonstrated that means have greater power than bias-corrected medians in most situations, which would make them the preferred choice.
