# Supplementary material for: Unnecessary reliance on multilevel modelling to analyse nested data in neuroscience: When a traditional summary-statistics approach suffices
Source: Curr Res Neurobiol. 2021 Nov 17;2:100024. doi: 10.1016/j.crneur.2021.100024 (PMC9559079; doi:10.1016/j.crneur.2021.100024)
Supplement: Multimedia component 1 [file mmc1.pdf]

Full title: Unnecessary reliance on multilevel modelling to analyse nested data in neuroscience: When a traditional summary-statistics approach suffices

Short title: Summary-statistics approach for nested data

Carolyn Beth McNabb[a] & Kou Murayama[a,b,c]

[a]School of Psychology and Clinical Language Sciences, University of Reading, Early Gate, Reading, RG6 7BE, United Kingdom

[b]Hector Research Institute of Education Sciences and Psychology, University of Tübingen, Europastraße 6, 72072 Tübingen, Germany

[c]Research Institute, Kochi University of Technology, Tosayamada, Kami City, 782-8502, Kochi, Japan

Corresponding authors:

Carolyn Beth McNabb, School of Psychology and Clinical Language Sciences, University of Reading, Early Gate, Reading, RG6 7BE, United Kingdom - c.b.mcnabb@reading.ac.uk

Kou Murayama, Hector Research Institute, University of Tübingen, Germany - k.murayama@uni-tuebingen.de

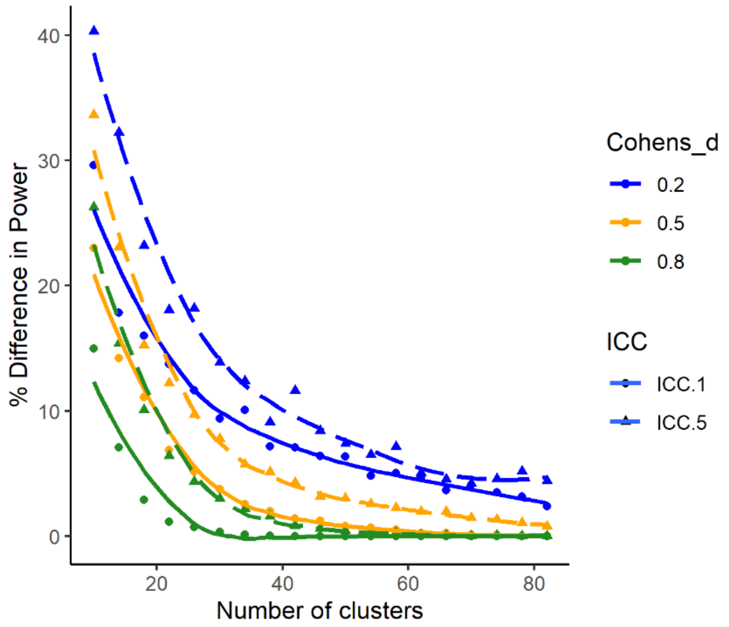

*Figure S1. Replication of Aarts et al. (2015) data. Power loss associated with use of multilevel modelling using log-likelihood estimation, including all 10000 simulations (including those resulting in singular fit or convergence errors). Power loss was calculated using the formula [(Power$_{MLM}$-Power$_{Summary\text{-}statistics\ approach}$)/Power$_{MLM}$]*100.*

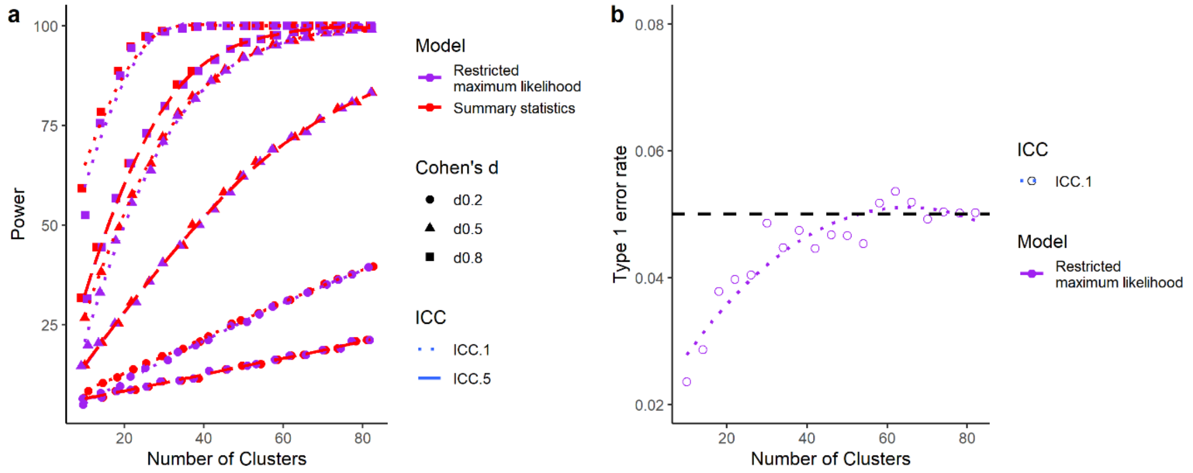

Figure S2. Power and associated Type I error rate with use of multilevel modelling with restricted maximum likelihood estimation (REML). a) Power is shown for all 10000 simulations for the summary statistics approach (red) and for those simulations that resulted in no singular fit or convergence errors for REML (purple). b) Type I error rate for multilevel modelling using REML (ICC = .1 only) for those simulations that resulted in no singular fit or convergence errors. Data are shown for small (circle), medium (triangle) and large (square) effect sizes (Cohen's d) and intra-class correlation (ICC) corresponding to the majority of total variance being due to within-group variance (ICC = .1, dotted line) and more total variance being due to between-group variance (ICC = .5, dashed line).

Table S1. Equivalence of multilevel model and summary statistics approaches for analysing data with unequal cluster sizes. Cluster sizes for Dataset C were between 8 and 12 observations; cluster sizes per condition for Dataset D (noting that each cluster had data for both conditions) were between 4 and 8 observations.

	**Dataset C**	**Dataset D**
Nesting description	Clusters within conditions	Conditions within clusters
**Multilevel model**	Intensity ~ Condition + (1 | Cluster)	Intensity ~ Condition + (1 + Condition | Cluster)
Fixed effects (SE)		
Intercept	.74 (.25)	.68 (.14)
	$t(10.2) = 3.02$ $p = .013$	$t(12.1) = 4.77$, $p = .0004$
Slope	.95 (.35)	.49 (.20)
	**$t(10.2) = 2.73$, $p = .021$**	**$t(9.5) = 4.36$, $p = .0016$**
Random effects		
Intercept variance	.32	.15
Slope variance		.13
Correlation		.20
Within cluster (residual) variance	.47	.57
**Summary statistics approach**		
Two sample *t*-test (equal variance)	**$t(10) = 2.77$, $p = .020$**	
Paired samples *t*-test		**$t(11) = 4.29$, $p = .0013$**
