# Supplementary material for: Unnecessary reliance on multilevel modelling to analyse nested data in neuroscience: When a traditional summary-statistics approach suffices
Source: Curr Res Neurobiol. 2021 Nov 17;2:100024. doi: 10.1016/j.crneur.2021.100024 (PMC9559079; doi:10.1016/j.crneur.2021.100024)
Supplement: Multimedia component 2 [file mmc2.pdf]

Peer Review Overview

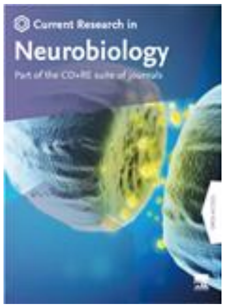

Manuscript Title: Unnecessary reliance on multilevel modelling to analyse nested data: when a traditional summary-statistics approach excels

Received	Apr 16, 2021
1st Decision	May 25, 2021
1st Revision Submitted	May 25, 2021
2nd Decision	Sep 03, 2021
2nd Revision Submitted	Oct 14, 2021
Accepted	Nov 08, 2021

1st Decision letter

Reference: CRNEUR-D-21-00019
Title: Unnecessary reliance on multilevel modelling to analyse nested data: when a traditional summary-statistics approach excels
Journal: Current Research in Neurobiology

Dear Dr McNabb,

Thank you for submitting your manuscript to Current Research in Neurobiology.

I have completed my evaluation of your manuscript. The reviewers recommend reconsideration of your manuscript following minor revision and modification. I invite you to resubmit your manuscript after addressing the comments below. Please resubmit your revised manuscript by Jun 24, 2021.

When revising your manuscript, please consider all issues mentioned in the reviewers' comments carefully: please outline every change made in response to their comments and provide suitable rebuttals for any comments not addressed. Please note that your revised submission may need to be re-reviewed.

Current Research in Neurobiology values your contribution and I look forward to receiving your revised manuscript.

CRNEUR aims to be a unique, community-led journal, as highlighted in the Editorial Introduction. As part of this vision, we will be regularly seeking input from the scientific community and encourage you and your co-authors to take the survey.

Kind regards,

Christopher I. Petkov
Editor in Chief
Current Research in Neurobiology

Comments from Editors and Reviewers:

Review of your paper has hit a major snag and I need to reject this version of the paper. The reviewer is correct that we need the code and data to replicate the results to be shared. Please correct and resubmit when ready. Then we can resume the review process.

Reviewer #1:

I am unable to review this paper as the authors have not made the code public on OSF. How, then, am I supposed to verify what they have done? They have wasted a lot of my time by doing this and it suggests they are not serious about the peer review. it also means I am simply unable to answer a lot of the questions above.

1st Author Response Letter

Response to comments from Editors and Reviewers:

Review of your paper has hit a major snag and I need to reject this version of the paper. The reviewer is correct that we need the code and data to replicate the results to be shared. Please correct and resubmit when ready. Then we can resume the review process.

Thank you for the opportunity to resubmit – this issue has now been resolved.

Comments from Reviewer 1

I am unable to review this paper as the authors have not made the code public on OSF. How, then, am I supposed to verify what they have done? They have wasted a lot of my time by doing this and it suggests they are not serious about the peer review. it also means I am simply unable to answer a lot of the questions above.

I am very sorry for this oversight. I have made the OSF page public. I did add one of the reviewers to the project when they requested access but perhaps this did not work as it should have. Please accept my sincere apology. I did not wish to waste anybody's time.

2nd Decision letter

Reference: CRNEUR-D-21-00019
Title: Unnecessary reliance on multilevel modelling to analyse nested data: when a traditional summary-statistics approach excels
Journal: Current Research in Neurobiology

Dear Dr McNabb,

Thank you for submitting your manuscript to Current Research in Neurobiology.

We have completed our evaluation of your manuscript. The reviewers recommend reconsideration of your manuscript following minor revision and modification. I invite you to resubmit your manuscript after addressing the comments below. Please resubmit your revised manuscript by Oct 03, 2021.

When revising your manuscript, please consider all issues mentioned in the reviewers' comments carefully: please outline every change made in response to their comments and provide suitable rebuttals for any comments not addressed. Please note that your revised submission may need to be re-reviewed.

Current Research in Neurobiology values your contribution and I look forward to receiving your revised manuscript.

CRNEUR aims to be a unique, community-led journal, as highlighted in the Editorial Introduction. As part of this vision, we will be regularly seeking input from the scientific community and encourage you and your co-authors to take the survey.

Kind regards,

Anna S Mitchell, Ph.D.
Editor in Chief
Current Research in Neurobiology

Comments from Editors and Reviewers:

Reviewer #1:

Thanks for making the simulation code available. I have now had a chance to review this paper and go through the simulations. I understand what the authors have done, and the methods seem to be appropriate, though I have questions about the degrees of freedom approximation for the mixed model approach.

Overall
This paper deals with the comparison between the mixed models approach (using all the data points as the units of analysis) and the summary statistics approach (using the animals or individuals as units of analysis). It points out the deep equivalences between using all the data points and having a random effect of individual; averaging all the data points from an individual; and doing a meta-analysis across individuals (where appropriate). This is helpful from a conceptual point of view, though the points are not exactly new. Indeed, one of the recommendations I often make to my students if using mixed models is to also run the analyses another way (e.g. summary statistics), just to understand what their numbers mean and how they relate to one another.

Given that mixed models can deal with a number of situations that summary stats cannot easily do (crossed random effects, unequal numbers of observations, designs where not all treatments are in all individuals, multivariate models with both between and within subjects predictors), and are equivalent in cases where there is a simple summary stats equivalent like the t-test or paired t-test, you might think that the answer would always be to use mixed models. However, the authors argue

that this might not be the case, since mixed models can suffer singular fit errors, and also inflation of type-I error rates depending on how significance is evaluated. Here I was less convinced, for reasons I discuss below.

Figure 4 is a useful summary of the authors' overall recommendations. However, it misses out some really important possibilities. There might be many predictors, including some that vary within clusters and some between (or perhaps some that vary within AND between). You simply can't deal with this using summary stats approaches, which are really only suitable for some very simple designs with a few predictors that are wholly between clusters, or one predictor that is wholly within cluster, so that you can do a paired t-test. Those designs are increasingly common and mixed models are really your only option; the simple summary stats option does not exist. Similarly, if the underlying observations are counts, or one-zero, you a better of using a generalized linear mixed model that reflects the true data generating process. That is not accounted for in figure 4.

Moreover, I was left with the feeling that all branches in figure 4 could lead to mixed models anyway. The right-hand branch, where it leads to using the summary statistics approach, you could also and equivalently use linear mixed models (which additionally give you all kinds of advantages like estimation of variance components, decompostion of effects into within and between subjects components, and estimating individual consistency). The middle branch, convergence and fit errors can often be eliminated by tweaking the model (see comment below), in which case you end up at mixed models anyway. So I would still recommend students to learn mixed models as this is the most general and flexible framework for data analysis (though, I concede, summary statistics are incredibly useful for *communicating* and *visualizing* results, even if the underlying analysis is mixed models). So, in as much as the paper is arguing against the widespread adoption of mixed modelling, I remain unconvinced. Understanding what mixed models are doing and how they relate to summary statistics (including when they come to the same thing) is however extremely valuable.

Anyway, these are just my views. Data analysis is to some extent a matter of taste, and of whatever is simplest and most effective to enable others to see your effects, but it is really important to understand how different approaches and their conclusions relate to one another, which this paper does help with. In addition to my general reflections above, I felt their were the following specific issues that needed attention.

Specific comments

1. The authors don't do a good job in tackling the question of what the right df are for significance tests in linear mixed models. Their simulations appear to use Satterthwaite's method via the lmerTest package. However, the paper does not say this explicitly, ann the short footnote on df in mixed models that is given is actively misleading on this point. There is a large literature on the different approaches to estimating df. These are known to have issues of inflated type I error rates in some cases under some circumstances. There is unfortunately no option but to go into this, and the authors need to explain what the issue is, what the options are, what lmerTest does, and what difference it makes (see Luke 2017, Peng and Redden 201, Kuznetsova et al. 2017). This interacts with the comparison of power and type-I error rates to the summary statistics approach.

2. The authors point out singular fit issues as a major issue in mixed models, and suggest that you basically can't use the method if you get these issues. However, these are often due to a near-zero amount of variance explained by a random effect. Under these circumstances, many authorities

consider it justified to remove this random effect. Indeed, many suggest that you can use information-theoretic criteria to pare down random effects if they have near-zero variances (see Alan Zuur's book, for example), prior to testing the fixed effects. This tends to reduce singular fit problems, but more importantly, it means that mixed models can under some circumstances be considerably more powerful than the summary stats approach. In short, if you can show that some level of clustering is unimportant in explaining variation, because data points within clusters are as good as independent, then your true n is higher than the number of clusters. I know this is an area of some disagreement (whether you always have to fit the maximal random effects, or whether you can use the data to reduce them), but the authors do need to consider and treat it more careful. By the way, convergence issues in R mixed models can often be fixed by a simple change of optimizer (e.g. control = lmerControl(optimizer ="Nelder_Mead"), or via optimx package and 'control = lmerControl(optimizer ='optimx', optCtrl=list(method='L-BFGS-B'))'.

3. Relatedly, for the 'conditions within clusters' situation, many people in my experience fit the random intercept for cluster but not the random slope. This does increase power, though I can see that it is philosophically not correct, since it assumes that if an individual responds to a treatment multiple times, they do so independently each time. However, some people would justify this by showing that the AIC was no smaller with the random slopes in. The authors need to consider under what circumstances it might be ok not to include the random slope, and what difference this makes to their arguments. It has the potential to make the mixed model approach more powerful than the number of animals. Also, it is worth explaining why the random slope should be the default in these cases - I myself had not really understood this until quite recently.

4. There are undiscussed issues with the summary stats approach. For example, do you take the mean for each animal? You could also take the median. In behavioural data, for example latencies, I have often found this useful because their might be some trials where the animal went to sleep or something, and the median is a better representation of their typical performance than the mean is. So that is an advantage of the summary statistics approach. On the other hand, this is a source of researcher degrees of freedom: there are several different summary stats one could use (and some people do all kinds of normalization at the level of the individual animal too), leading to multiple possible analyses and the temprtation to use the one that produces the nicest result.

5. A further advantage of learning mixed models over the kinds of summary stats approaches described here is that they are better developed for non-Gaussian cases. For example, if you are interested in the number of times an animal does something in a three minute period, you are almost certainly better off using a mixed model of the Poisson family and using each observation as a unit of analysis, with a random effect of individual, than you are just talking the per animal means and doing a t-test. Why? Because the underlying data are counts, are bounded at zero, and really don't satisfy the assumptions of classical tests like the t-test, even though after averaging by individual it might look like they do. Learning mixed models allows the student to switch to Poisson or Bernouilli type cases as required, with only a mimimal change of model, in a way that allows them to be much more faithful to the true data generating process.

6. Relatedly, very often we are interested in the effects of a whole lot of predictors, some of which vary within clusters, and some between. If we don't have completely designed experiment (for example, complex experiments, experiments where the data you can get are limited for practical reasons, fieldwork or epidemiology), then not all variables are equally distributed across clusters, and this can't be eliminated by design. For these cases you just have to do some kind of multilevel model.

The authors seem to have only a narrow range of possible cases in mind in figure 4.

7. It would be really helpful if the authors would reference the terms 'within-subjects design' and 'between-subjects designs' in relation to conditions-within-clusters and clusters-within-conditions, as these are familiar terms to many psychologists.

Luke, S. G. (2017). Evaluating significance in linear mixed-effects models in R. Behavior Research Methods, 49(4), 1494-1502. https://doi.org/10.3758/s13428-016-0809-y
Li, P., & Redden, D. T. (2015). Comparing denominator degrees of freedom approximations for the generalized linear mixed model in analyzing binary outcome in small sample cluster-randomized trials. BMC Medical Research Methodology, 15(1), 1-12. https://doi.org/10.1186/s12874-015-0026-x
Kuznetsova, A., Brockhoff, P. B., & Christensen, R. H. B. (2017). lmerTest Package: Tests in Linear Mixed Effects Models. Journal of Statistical Software, 82(13). https://doi.org/10.18637/jss.v082.i13

Reviewer #2:

The manuscript of McNabb & Murayama targets the neuroscience community and describes a simpler statistical approach to analyze experimental designs where units of observations are nested within clusters of equal size. The authors present computational simulations to emphasize that the the "summary-statistics" approach comprising two steps: 1) summarising the data within each cluster by the cluster mean; and 2) applying a t-test to these cluster
means is an equally valid but simpler approach to REML estimation in a mixed model. Summary-statistics approach avoids frequent computational problems during the estimation of covariance parameters in linear mixed models (LMM) encountered in practice. Authors' case is presented well and is easier to understand for practitioners than other methods to troubleshoot the model fitting in LMMs, such as fitting an implied marginal model, or adjusting for downward bias in the variance of fixed effects by Kenward-Roger method.

I have only minor suggestions:

In section 4 where dealing with moderate inequality in cluster size is discussed, it would be useful to mention the case where cluster sizes become unequal due to missing observations. LMMs are praised for their ability to handle missing data, therefore I wonder how the sufficient summary-statistics approach would deal with differences in cluster size due to the missing observations.

Furthermore, and importantly, the conditions under which multilevel
models outperform simple statistical methods (such as the t-test) and the associated gains in power reported in previous influential literature4, 5 are based on inappropriately conducted statistical simulations.

This sentence would benefit for a brief description of an error made during the statistical simulation

7. Murayama, K., Usami, S. & Sakaki, M. (2020).

Reference #7 is not fully specified.
The style of some other references needs correction.

2nd Author Response Letter

Response to comments from Editors and Reviewers:

Thank you for the opportunity to revise our manuscript “Unnecessary reliance on multilevel modelling to analyse nested data: when a traditional summary-statistics approach excels” for consideration in Current Research in Neurobiology. We appreciate the time and consideration that went into the review of our manuscript and have done our best to address each of the reviewers’ concerns below. Changes made following review are highlighted in yellow in the main document. We refer to page numbers where relevant. Thank you again for the opportunity to revise our work. Please do let us know if there is anything else you require from us.

Comments from Reviewer 1

Thanks for making the simulation code available. I have now had a chance to review this paper and go through the simulations. I understand what the authors have done, and the methods seem to be appropriate, though I have questions about the degrees of freedom approximation for the mixed model approach.

Overall

This paper deals with the comparison between the mixed models approach (using all the data points as the units of analysis) and the summary statistics approach (using the animals or individuals as units of analysis). It points out the deep equivalences between using all the data points and having a random effect of individual; averaging all the data points from an individual; and doing a meta- analysis across individuals (where appropriate). This is helpful from a conceptual point of view, though the points are not exactly new. Indeed, one of the recommendations I often make to my students if using mixed models is to also run the analyses another way (e.g. summary statistics), just to understand what their numbers mean and how they relate to one another.

Given that mixed models can deal with a number of situations that summary stats cannot easily do (crossed random effects, unequal numbers of observations, designs where not all treatments are in all individuals, multivariate models with both between and within subjects predictors), and are equivalent in cases where there is a simple summary stats equivalent like the t-test or paired t-test, you might think that the answer would always be to use mixed models. However, the authors argue that this might not be the case, since mixed models can suffer singular fit errors, and also inflation of type-I error rates depending on how significance is evaluated. Here I was less convinced, for reasons I discuss below.

Figure 4 is a useful summary of the authors' overall recommendations. However, it misses out some really important possibilities. There might be many predictors, including some that vary within clusters and some between (or perhaps some that vary within AND between). You simply can't deal with this using summary stats approaches, which are really only suitable for some very simple designs with a few

predictors that are wholly between clusters, or one predictor that is wholly within cluster, so that you can do a paired t-test. Those designs are increasingly common and mixed models are really your only option; the simple summary stats option does not exist. Similarly, if the underlying observations are counts, or one-zero, you a better of using a generalized linear mixed model that reflects the true data generating process. That is not accounted for in figure 4.

** We thank the reviewer for their comments – as the reviewer points out, figure 4 (i.e. flowchart of the choice of different methods) did not cover all possible scenarios and so to eliminate confusion, we have removed this figure from the manuscript. With regard to the type of data that can be analysed with the summary-statistics approach, when the predictor is binary and data are balanced, the equivalence between the summary-statitics approach and multilevel modelling holds regardless of the level of predictors and number of predictors (for mathematical proof, see (Murayama et al., in press)). However, it is true that the equivalence does not always hold in case of continuous predictors and/or non-linear link function. The main focus of the current manuscript is on the most simple case (Figure 1) because (1) previous influential papers (i.e. (Aarts et al., 2015; Aarts et al., 2014)) wrongly claimed the advantage of mixed-effects modelling with these designs and (2) this is still a common experimental design in neuroscience. However, we have expanded on section 4 discussing complicated designs in relation to sufficient summary-statistics approach (page 12) and created a new section (section 5, page 12) describing designs for which the summary-statistics approach is inappropriate – we now refer to this section in our introduction (page 4).*

Moreover, I was left with the feeling that all branches in figure 4 could lead to mixed models anyway. The right-hand branch, where it leads to using the summary statistics approach, you could also and equivalently use linear mixed models (which additionally give you all kinds of advantages like estimation of variance components, decompostion of effects into within and between subjects components, and estimating individual consistency). The middle branch, convergence and fit errors can often be eliminated by tweaking the model (see comment below), in which case you end up at mixed models anyway. So I would still recommend students to learn mixed models as this is the most general and flexible framework for data analysis (though, I concede, summary statistics are incredibly useful for *communicating* and *visualizing* results, even if the underlying analysis is mixed models). So, in as much as the paper is arguing against the widespread adoption of mixed modelling, I remain unconvinced. Understanding what mixed models are doing and how they relate to summary statistics (including when they come to the same thing) is however extremely valuable.

** We agree that Figure 4 (i.e. flowchart of the choice of different methods) is misleading. As such, we have decided to omit Figure 4 and focus on the comparison of the two approaches. We believe that by focusing on the comparison, the paper also serves as a good educational material to understand mixed-effects modelling, as the reviewer indicated.*

Anyway, these are just my views. Data analysis is to some extent a matter of taste, and of whatever is simplest and most effective to enable others to see your effects, but it is really important to understand how different approaches and their conclusions relate to one another, which this paper does help with.

** We appreciate the reviewer's positive comments with regard to the utilty of this manuscript as well as their insightful, constructive comments regarding suggested improvements to our work. We have done our best to address each of the reviewer's concerns (detailed below).*

In addition to my general reflections above, I felt their were the following specific issues that needed attention.

Specific comments

1. The authors don't do a good job in tackling the question of what the right df are for significance tests in linear mixed models. Their simulations appear to use Satterthwaite's method via the lmerTest package. However, the paper does not say this explicitly, ann the short footnote on df in mixed models that is given is actively misleading on this point. There is a large literature on the different approaches to estimating df. These are known to have issues of inflated type I error rates in some cases under some circumstances. There is unfortunately no option but to go into this, and the authors need to explain what the issue is, what the options are, what lmerTest does, and what difference it makes (see Luke 2017, Peng and Redden 201, Kuznetsova et al. 2017). This interacts with the comparison of power and type-I error rates to the summary statistics approach.

** When the data are balanced (this is the case for our simulation), we believe the degrees freedom (and estimated standard errors) are always the same as indicated in the original manuscript, regardless of whether one uses Satterthwaite's method, Kenward-Roger's method, or the containment method (see(Murayama et al., in press), which touches on the issue). This is the case even if the sample size is small. Therefore, our conclusion from the simulation is unchanged. Note that this does not mean that the issue does not exist with a balanced design --- the underestimation of standard errors should occur in theory (see McNeish, 2017) but this cannot be resolved by these correction methods as the methods can only account for the potential bias due to the design.*

However, these correction methods make a difference when the design is unbalanced and/or predictors are continuous. We clarified this point in the footnote and detail the methods used for our own simulations on page 7.

2. The authors point out singular fit issues as a major issue in mixed models, and suggest that you basically can't use the method if you get these issues. However, these are often due to a near-zero amount of variance explained by a random effect. Under these circumstances, many authorities consider it justified to remove this random effect. Indeed, many suggest that you can use information-theoretic criteria to pare down random effects if they have near-zero variances (see Alan Zuur's book, for example), prior to testing the fixed effects. This tends to reduce singular fit problems, but more importantly, it means that mixed models can under some circumstances be considerably more powerful than the summary stats approach. In short, if you can show that some level of clustering is unimportant in explaining variation, because data points within clusters are as good as independent, then your true n is higher than the number of clusters. I know this is an area of some disagreement (whether you always have to fit the maximal random effects, or whether you can use the data to reduce them), but the authors do need to consider and treat it more careful.

** As the reviewer correctly pointed out, we believe this is still a matter of debate. It is true that such strategy ensures high statistical power, but many also indicated that the misspecification of random variance structure would increase Type-1 error rates (Barr et al., 2013; Ferron et al., 2002; Hoffman, 2015) (Barr et al., 2013; Hoffman, 2015). When there is such a tradeoff, normally researchers are encouraged to give priority to controling for Type-1 error rates as much as possible. In addition, neuroscience research tends to have small sample size and in that case, singular fit error is likely to occur*

due to the unstable estimates of random effects (not the true zero variance). Given that researchers are motivated to obtain a significant effect (p hacking) and the tendency to use small sample size in the field, we are hesitant to encourage researchers to remove random effects in the paper (we must admit that we do that in our work when there is really no other solution --- nevertheless, we are hesitant to encourage researchers). That being said, it is true that we did not discuss the debate in the original manuscript --- we have extended the section by incorporating the controversy on page 9 (also in footnote).

By the way, convergence issues in R mixed models can often be fixed by a simple change of optimizer (e.g. control = lmerControl(optimizer ="Nelder_Mead"), or via optimx package and 'control = lmerControl(optimizer ='optimx', optCtrl=list(method='L-BFGS-B'))'.

**We thank the reviewer for this very helpful tip. We have added it as a footnote in the manuscript (page 9).*

3. Relatedly, for the 'conditions within clusters' situation, many people in my experience fit the random intercept for cluster but not the random slope. This does increase power, though I can see that it is philosophically not correct, since it assumes that if an individual responds to a treatment multiple times, they do so independently each time. However, some people would justify this by showing that the AIC was no smaller with the random slopes in. The authors need to consider under what circumstances it might be ok not to include the random slope, and what difference this makes to their arguments. It has the potential to make the mixed model approach more powerful than the number of animals. Also, it is worth explaining why the random slope should be the default in these cases - I myself had not really understood this until quite recently.

** As indicated above, our opinion is that we should always include random slopes to avoid the risk of Type-1 error rate inflation. Model selection (e.g. using AIC) could help but does not completely eliminate the risk. Again, we have discussed the issue (including the fact that there is still controversy) in more depth in the revised manuscript (page 10).*

4. There are undiscussed issues with the summary stats approach. For example, do you take the mean for each animal? You could also take the median. In behavioural data, for example latencies, I have often found this useful because their might be some trials where the animal went to sleep or something, and the median is a better representation of their typical performance than the mean is. So that is an advantage of the summary statistics approach. On the other hand, this is a source of researcher degrees of freedom: there are several different summary stats one could use (and some people do all kinds of normalization at the level of the individual animal too), leading to multiple possible analyses and the temprtation to use the one that produces the nicest result.

**The reviewer makes a good point. This flexibility is actually an advantage of the summarystatistics approach --- "summary-statistics" does not always mean the average; however, we share their concern about researcher degrees of freedom. We have made a note of these issues at the end of section 3 (page 10), recommending that researchers report their methods in a transparent and reproducible manner. We have included further information on the controversy over the use of the median summary statistic for skewed data in the footnote of page 10 to aid researchers when making these decisions.*

5. A further advantage of learning mixed models over the kinds of summary stats approaches described here is that they are better developed for non-Gaussian cases. For example, if you are interested in the

number of times an animal does something in a three minute period, you are almost certainly better off using a mixed model of the Poisson family and using each observation as a unit of analysis, with a random effect of individual, than you are just talking the per animal means and doing a t-test. Why? Because the underlying data are counts, are bounded at zero, and really don't satisfy the assumptions of classical tests like the t-test, even though after averaging by individual it might look like they do. Learning mixed models allows the student to switch to Poisson or Bernouilli type cases as required, with only a mimimal change of model, in a way that allows them to be much more faithful to the true data generating process.

** We agree --- we have added the discussion to the new section (section 5, page 12) on complex models.*

6. Relatedly, very often we are interested in the effects of a whole lot of predictors, some of which vary within clusters, and some between. If we don't have completely designed experiment (for example, complex experiments, experiments where the data you can get are limited for practical reasons, fieldwork or epidemiology), then not all variables are equally distributed across clusters, and this can't be eliminated by design. For these cases you just have to do some kind of multilevel model. The authors seem to have only a narrow range of possible cases in mind in figure 4.

** As noted, we have created a new section (section 5) discussing such a design in relation to sufficient summary-statistics approach. We have also omitted Figure 4.*

7. It would be really helpful if the authors would reference the terms 'within-subjects design' and 'between-subjects designs' in relation to conditions-within-clusters and clusters-within-conditions, as these are familiar terms to many psychologists.

**We have added a sentence to the first paragraph (page 3) to explain the relationship between these terms.*

Luke, S. G. (2017). Evaluating significance in linear mixed-effects models in R. Behavior Research Methods, 49(4), 1494-1502. https://doi.org/10.3758/s13428-016-0809-y

Li, P., & Redden, D. T. (2015). Comparing denominator degrees of freedom approximations for the generalized linear mixed model in analyzing binary outcome in small sample cluster-randomized trials. BMC Medical Research Methodology, 15(1), 1-12. https://doi.org/10.1186/s12874-015-0026-x

Kuznetsova, A., Brockhoff, P. B., & Christensen, R. H. B. (2017). lmerTest Package: Tests in Linear Mixed Effects Models. Journal of Statistical Software, 82(13). https://doi.org/10.18637/jss.v082.i13

Comments from Reviewer 2

The manuscript of McNabb & Murayama targets the neuroscience community and describes a simpler statistical approach to analyze experimental designs where units of observations are nested within clusters of equal size. The authors present computational simulations to emphasize that the the "summary-statistics" approach comprising two steps: 1) summarising the data within each cluster by the cluster mean; and 2) applying a t-test to these cluster means is an equally valid but simpler approach to REML estimation in a mixed model. Summarystatistics approach avoids frequent computational

problems during the estimation of covariance parameters in linear mixed models (LMM) encountered in practice. Authors' case is presented well and is easier to understand for practitioners than other methods to troubleshoot the model fitting in LMMs, such as fitting an implied marginal model, or adjusting for downward bias in the variance of fixed effects by Kenward-Roger method.

**We thank the reviewer for taking the time to review our work. We have addressed each of the suggestions below.*

I have only minor suggestions:

In section 4 where dealing with moderate inequality in cluster size is discussed, it would be useful to mention the case where cluster sizes become unequal due to missing observations. LMMs are praised for their ability to handle missing data, therefore I wonder how the sufficient summarystatistics approach would deal with differences in cluster size due to the missing observations.

** We have mentioned this in a revised manuscript on page 11.*

Furthermore, and importantly, the conditions under which multilevel models outperform simple statistical methods (such as the t-test) and the associated gains in power reported in previous influential literature4, 5 are based on inappropriately conducted statistical simulations.

This sentence would benefit for a brief description of an error made during the statistical simulation

**We have specified that the error is due to the incorrect choice of method for standard error estimation. We go into more detail regarding this issue in section 2 and so have referred readers there for more detail.*

7. Murayama, K., Usami, S. & Sakaki, M. (2020).

Reference #7 is not fully specified.

The style of some other references needs correction.

** Thank you for pointing these out – we have now corrected the references.*

References

Aarts, E., Dolan, C.V., Verhage, M., van der Sluis, S., 2015. Multilevel analysis quantifies variation in the experimental effect while optimizing power and preventing false positives. BMC neuroscience 16, 94.

Aarts, E., Verhage, M., Veenvliet, J.V., Dolan, C.V., Van Der Sluis, S., 2014. A solution to dependency: using multilevel analysis to accommodate nested data. Nature neuroscience 17, 491.

Barr, D.J., Levy, R., Scheepers, C., Tily, H.J., 2013. Random effects structure for confirmatory hypothesis testing: Keep it maximal. Journal of memory and language 68, 10.1016/j.jml.2012.1011.1001.

Ferron, J., Dailey, R., Yi, Q., 2002. Effects of Misspecifying the First-Level Error Structure in Two-Level Models of Change. Multivariate Behavioral Research 37, 379-403.

Hoffman, L., 2015. Longitudinal Analysis. Routledge.

Murayama, K., Usami, S., Sakaki, M., in press. A simple and easy method for power analysis in mixedeffects modelling with nested data: Just a t value often suffices. Psychological Methods.

Accept Letter

Dear Dr McNabb,

Thank you for submitting your manuscript to Current Research in Neurobiology. I am pleased to inform you that your manuscript has been accepted for publication. Additional reviewer comments are appended below.

Your accepted manuscript will now be transferred to our production department. We will create a proof which you will be asked to check, and you will also be asked to complete a number of online forms required for publication. If we need additional information from you during the production process, we will contact you directly.

We appreciate and value your contribution to Current Research in Neurobiology. We regularly invite authors of recently published manuscript to participate in the peer review process. If you were not already part of the journal's reviewer pool, you have now been added to it. We look forward to your continued participation in our journal, and we hope you will consider us again for future submissions.

CRNEUR aims to be a unique, community-led journal, as highlighted in the Editorial Introduction. As part of this vision, we will be regularly seeking input from the scientific community and encourage you and your co-authors to take the survey.

Kind regards,

Yogita Chudasama
Associate Editor
Current Research in Neurobiology

Editor and Reviewer comments:

Reviewer 1: Thanks for the revised version. The authors have responded well and constructively, and I have no further comments. It's a useful contribution to the literature.

-------- End of Review Comments --------
